# Oral anticoagulation in very elderly patients with atrial fibrillation: Results from the prospective multicenter START2-REGISTER study

**DOI:** 10.1371/journal.pone.0216831

**Published:** 2019-05-23

**Authors:** Daniela Poli, Emilia Antonucci, Walter Ageno, Lorenza Bertù, Ludovica Migliaccio, Lucia Martinese, Giuseppe Pilato, Sophie Testa, Gualtiero Palareti

**Affiliations:** 1 Center for Atherothrombotic Diseases, Azienda Ospedaliero-Universitaria Careggi, Firenze, Italy; 2 Arianna Anticoagulazione Foundation, Bologna, Italy; 3 Department of Medicine and Surgery, University of Insubria, Varese, Italy; 4 Department of Experimental and Clinical Medicine, University of Florence, Firenze, Italy; 5 Haemostasis and Thrombosis Centre, Hospital of Cremona, Cremona, Italy; Universita degli Studi di Napoli Federico II, ITALY

## Abstract

Direct oral anticoagulants (DOACs) have shown similar efficacy and safety with respect to warfarin in patients with atrial fibrillation (AF). However, the proportion of patients aged ≥85 years enrolled in clinical trials was low and the applicability of their results to very elderly patients is still uncertain. We have carried out a prospective cohort study on AF patients aged ≥85 years enrolled in the Survey on anticoagulaTed pAtients RegisTer (START2-Register) and treated with either VKAs or DOACs, with the aim to evaluate mortality, bleeding and thrombotic rates during a long-term follow-up. We enrolled 1124 patients who started anticoagulation at ≥85 years with VKA (58.7%) or DOACs (41.3%), Clinical characteristics of patients were similar, except for a higher prevalence of coronary artery disease and renal failure in VKAs patients and of a history of previous bleeding and previous stroke/TIA in patients on DOACs. Median CHA_2_DS_2_VASc and HAS-BLED scores were similar between the two groups. During follow-up, 47 major bleedings (rate 2.3 x100 pt-yrs) and 19 stroke/TIA (0.9 x100 pt-yrs) were recorded. The incidence of bleeding was similar between patients on VKAs and DOACs. Patients on DOACs showed a higher rate of thrombotic events during treatment (rate 1.84 and 0.50,respectively). Mortality rate was higher in patients on VKAs than in patients on DOACs (HR 0.64 (95% CI 0.46–0.91). In conclusion, we confirm the overall safety and effectiveness of anticoagulant treatment in very elderly AF patients, with lower mortality rates in DOACs patients, similar bleeding risk, and a higher risk for cerebral thrombotic events in DOACs patients.

## Introduction

Oral anticoagulant treatment with either vitamin K antagonists (VKAs) or direct oral anticoagulants (DOACs) is recommended for the prevention of stroke or systemic embolism and all-cause mortality in patients with atrial fibrillation (AF) [1]. DOACs have shown similar efficacy and safety with respect to warfarin [2], at fixed dosages reducing the inconvenience of dose adjustment required by VKAs. Nowadays, DOACs are the preferred treatment option in some guidelines [1].

The median age of patients enrolled in clinical trials with DOACs in AF patients ranged from 70 to 73 years. Given the relatively young mean age of the study populations, a post-hoc analysis of these randomized clinical trials focusing on patients aged >75 years [3] was carried out and confirmed the efficacy and safety of the DOACs also in these patients. However, the proportion of patients aged 80 years or older was low and, therefore, the applicability of the results of these trials to very elderly patients is still a matter of debate. Additional data on the safety of the DOACs in very elderly patients comes from retrospective observational studies only [4, 5]. More information is available from studies on elderly patients treated with VKAs [6–9], which showed an acceptably low bleeding risk even in advanced classes of age, with a favorable net clinical benefit of the treatment [5, 10]. Because of the need for additional evidence on the safety and effectiveness of the DOACs in very elderly AF patients, we have carried out a prospective cohort study on AF patients aged ≥ 85 years enrolled in the Survey on anticoagulaTed pAtients RegisTer (START2-Register) and treated with either VKAs or DOACs, aiming at evaluating the bleeding and thrombotic complications rates and mortality during a long-term follow-up.

## Methods

The START2-Register is an observational, multicenter, prospective cohort study that includes adults (> 18 years) who start anticoagulation therapy, whatever the clinical indication for the therapy, the drug and dosage used [11]. The aim of the START2-Register is to collect data on effectiveness and safety of anticoagulant treatments, on determinants of adverse events in anticoagulated patients, as well as on their quality of life and compliance to treatment. The registry has been approved on October 2011 (N = 142/2010/0/0ss) by the Ethical Committee of the Institution of the Coordinating Member (G.P.) (AziendaOspedaliero-Universitaria, Policlinico S. Orsola-Malpighi, Bologna, Italy), and is registered in ClinicalTrials.gov Identifier: NCT02219984. The participants gave their written informed consent. The study is ongoing and actively recruiting. For the purpose of the present analysis, data were collected from January 2012 to April 2018. We here present the results of the cohort of non-valvular atrial fibrillation (NVAF) patients who started the anticoagulation at the age ≥ 85 years.

All participating centers are asked to consecutively include patients who start anticoagulant treatment, for any indication and with any available drug, if this is planned to last for at least 3 months. Patients with life-expectancy < 6 months, or not residents in the participant region, or planning to leave in the next six months after enrolment are not eligible, as well as patients already enrolled in phase II or III clinical studies. Follow-up of enrolled patients is mandatory for at least one year, but is recommended to be indefinite. Participants are required to enroll their patients consecutively, without any a priori exclusion criteria other than life-expectancy or geographical inaccessibility. Definition of the time-frame for enrolment (e. g. one week every month, or the first month of the year) is left at each participant’s discretion, as long as it provides a random enrolment of patients. Baseline patient’s clinical features are recorded by participants on web-based case report forms (CRF) and include: demographic and clinical characteristics of patients, clinical indication for treatment, associated risk factors for thromboembolic complications or bleeding occurring during treatment, laboratory routine data, type of anticoagulant drug used and dose (or expected therapeutic range), use of concomitant drugs. For patients treated with VKA, all INR controls, the subsequent dosing prescriptions and information at each visit about possible clinical events and changes in the medical history are automatically captured via informatics. All INRs were recorded and time in therapeutic range (TTR) [12] of the last 6 months of treatment was reported. Participants are required to regularly follow-up all enrolled patients at least quarterly, by phone call or ambulatory visit. An ambulatory follow-up visit is mandatory at least annually. In the presence of severe dementia or frequent falls or bed rest the patients were defined frail. Creatinine clearance was calculated by the Cockroft-Gault formula [13]. AF patients are stratified for stroke risk evaluation according to CHA_2_DS_2_VASc [14] score, while baseline bleeding risk is evaluated by using HAS-BLED score [15].

Major endpoints of the study were first major bleeding (MB); stroke or transient ischemic attack (TIA) and death for all causes. MB were defined as recommended by the International Society on Thrombosis and Haemostasis [16]. Stroke was defined as a syndrome characterized by rapidly developing clinical symptoms and/or signs of focal and at times global loss of brain function, lasting >24 hours, not explained by other causes and in the absence of primary hemorrhage. Ischemic stroke was defined as a stroke with either a normal brain CT or evidence of a recent infarction in the clinically relevant area of the brain on a CT or MR scan within three weeks of the event, while previous TIA was diagnosed when neurological defects lasted >24 hours [17]. Death was defined as consequence of bleeding or of stroke/TIA; all the other causes were reported as ‘not related to anticoagulation’.

### Statistical analysis

Data were described as the mean value and standard deviation (SD) for continuous variables and proportions for categorical variables. Differences between continuous values were assessed using the unpaired t-test, categorical variables were compared by the Chi-square test or Fisher exact test as appropriate. The median and interquartile range (IQR) follow-up time were calculated and the median test applied to test difference between groups. The incidences of death, thrombotic accidents, major bleedings were calculated by dividing the number of events by person time at risk. The incidence rate ratio and together with the 95% confidence interval (95%CI) were calculated.

A propensity score was derived to model the probability of receiving different study medication (DOACs vs AVKs) as a function of the follow variables: age at enrollment, sex, diabetes, hypertension, Chronic Obstructive Pulmonary Disease (COPD), previous stroke, previous bleeding, frail subject, active cancer, renal failure (defined as GFR<30 ml/min), coronary artery disease (CAD).

The inverse probability of treatment weighted (IPTW) was used to create the pseudo-population in which the treatment is independent of the measured confounders.

To estimate the impact of treatment on death, thrombotic accident and major bleedings, after checking the proportional hazards assumption, survival analysis was performed using Cox regression model. Crude and weighted hazard ratios (HRs) together with the 95%CI were calculated. Cole and Hernan method to stabilized the weights was applied [18, 19].

When exploring the hazard of thrombotic accidents and major bleedings, death was treated as competing risk using the Fine and Gray competing–risk regression model.

Kaplan-Meier survival curves unadjusted and adjusted for IP-weighted approach were also provide.

For descriptive purpose, a multivariate hazard model was calculated for the three outcomes.

All analysis was carried out using SAS statistical package (Version 9.4 for Windows. SAS Institute Inc. Cary NC).

## Results

We enrolled 1124 patients who started anticoagulation at ≥85 years: 660 of them received AVK (58.7%) and 464 DOACs (41.3%); they were followed-up for a total period of 2037 patient-years (pt-yrs). Twenty-six patients (2.3%) were lost at follow-up. All patients on AVK were naïve to anticoagulation, whereas 142/464 patients (30.6%) on DOACs had been previously anticoagulated with AVK. Patients on DOACs were on treatment with dabigatran 83 (17.8%), apixaban 192 (41.4%), rivaroxaban 158 (34.1%) and edoxaban 31 (6.7%). Patients on VKAs showed a median TTR of 70% (IQR 58–78). Because in Italy DOACs were marketed since the summer of 2013, the median duration of follow-up of patients treated with DOACs was shorter (1.15 years) than the follow-up of patients on VKA (1.74 years). Baseline characteristics of patients are reported in Table 1.

**Table 1:** Patients with atrial fibrillation, 85 years or older treated with VKAs or DOACs. Descriptive statistics of baseline features by treatment group.

	All VKAs (N = 660)	DOACs naive (N = 322)		All DOACs (N = 464)	
	N%	N (%)	p-value*	N (%)	p-value†
Sex- Female	374(56.7)	196 (60.9)	0.21	265 (57.5)	0.77
Hb<10 gr/DL	31 (4.7)	10 (3.1)	0.24	15 (3.2)	0.22
Platelet<100000	5 (0.8)	4 (1.2)	0.45	5 (1.1)	0.57
***Co-morbidity***					
Renal failure (creatinine clearance<30)	163 (24.7)	33 (10.3)	**<0.0001**	40 (8.6)	**<0.0001**
Previous cancer	103 (15.6)	50 (15.5)	0.97	72 (15.5)	0.97
Active cancer	18 (2.7)	4 (1.2)	0.14	8 (1.7)	0.27
Diabetes mellitus	113 (17.1)	56 (17.4)	0.92	80 (17.2)	0.96
Hypertension	562 (85.2)	262 (81.4)	0.13	386 (83.2)	0.37
Previous stroke	106 (16.1)	80 (24.8)	**0.001**	109 (23.5)	**0.001**
Previous bleeding	17(2.6)	18 (5.6)	**0.02**	32 (6.9)	**0.001**
Coronary artery disease	147 (22.3)	44 (13.7)	**0.001**	63 (13.6)	**0.0002**
Heart Failure	202 (30.6)	89 (27.6)	0.34	130 (28.0)	0.35
POAD	64 (9.7)	27 (8.4)	0.51	36 (7.8)	0.26
BPCO	97 (14.7)	36 (11.2)	0.13	60 (12.9)	0.40
Frail subjects‡	61 (9.2)	36 (11.2)	0.34	57 (12.3)	0.10
Age (Years)—Mean (SD)	87.4 (2.2)	88.4 (2.8)	**<0.0001**^§^	88.2 (2.7)	**<0.0001**^§^
***Co-medication***					
Antiplatelet drugs	63 (9.5)	23 (7.1)	**0.21**	30 (6.5)	**0.07**
***Risk stratification scores***					
CHA_2_DS_2_VASc—Mean (SD)	4.4 (1.2)	4.5 (1.3)	0.44^§^	4.5 (1.3)	0.73^§^
HASBLED—Mean (SD)	2.4 (0.7)	2.3 (0.8)	0.08^§^	2.3 (0.7)	**0.03**^§^
Follow-up (Months)—Median (IQR)	20.8(31.8)	12.7(16.5)	**<0.0001**^||^	13.7 (16.0)	**<0.0001**^||^

VKA = vitamin k antagonist; DOAC = direct oral anticoagulant

*Chi-square or fisher exact test p-value. DOACs naive vs. VKAs; † Chi-square of Fisher exact p-value, All DOACs vs. VKAs

‡ Patient with dementia or bed rest or prone to fall

§ T-test p-value

|| Median test p-value

Clinical characteristics of patients were similar, except for a higher prevalence of coronary artery disease and of renal failure among patients on VKAs. Conversely, a history of previous bleeding and of previous stroke/TIA were more frequently represented among patients on DOACs. Median CHA_2_DS_2_VASc and HAS-BLED scores were similar between the two groups.

During follow-up, 47 major bleedings (rate 2.3 x100 pt-yrs) and 19 stroke/TIA (0.9 x100 pt-yrs) were recorded; 284/1124 (25.3%) of patients died, 12 because of bleeding and 2 because of stroke, all the other deaths were classified by the investigators as not related to anticoagulation. Among DOACs patients who had a major bleeding 3/16 (19%) died for this reason, one of them was also on treatment with low-dose aspirin. Among VKA patients with major bleeding, 9/31 (29%) had fatal hemorrhagic events, one patient was also on treatment with low-dose aspirin. We recorded 2 fatal stroke, one in a patient on treatment with edoxaban 30 mg od and one in a patient on VKA, none were on treatment with aspirin.

Among patients who did not experience any outcome event, anticoagulation was stopped due to personal choice or decision of the treating physician in 57/444 (12.8%) of VKAs patients (rate 4.1% pt-yrs) and 18/404 (4.5%) of DOACs patients (rate 2.8% pt-yrs) (RR 1.5; 0.9–2.7 95%CI p = 0.13).

The incidence of bleeding was similar between patients on VKAs or DOACs (Table 2).

**Table 2:** Events recorded during follow-up in patients 85 years or older with atrial fibrillation, treated with VKAs or DOACs, or DOACs treatment-naive.

	VKAs (N = 660)		DOACs all (N = 464)		DOACs naive only (N = 322)	
	N	ratex100 pt-yr (CI95%)	N	rate x100 pt-yrs (CI95%)	N	rate x100 pt-yrs (CI 95%)
Patient-yrs	1385		649		417	
Death	224	16.2 (14.2;18.5)	60	9.24 (7.25–11.72)	42	10.1 (6.35;15.95)
Thrombotic accidents	8	0.58 (0.29;1.16)	12	1.84 (1.06–3.20)	8	1.92 (0.33;11.1)
Major bleeding	31	2.24 (1.58;3.19)	16	2.46 (1.52–3.97)	11	2.64 (0.93; 7,84)
Cerebral bleeding	9	0.64 (0.34–1.23)	5	0.77 (0.33–1.79)	5	1.19 (0.51–2.78)
Gastrointestinal bleeding	8	0.86 (0.50–1.51)	7	2.00 (1.17–3.40)	5	1.19 (0.51–2.78)

VKA = vitamin k antagonist; DOAC = direct oral anticoagulant

At the multivariate competing risk analysis, the history of previous bleeding and of active cancer were significantly associated to bleeding risk (Table A in S1 File). Among patients on VKAs no difference in TTR was recorded in relation to occurrence of major bleeding. The rate of thrombotic events during treatment was higher in patients on DOACs than in those on VKAs (1.84% pt-yrs and 0.50, respectively; RR 3.2; 95%CI 1.20–9.02, p = 0.01). None of the considered risk factors for cerebral vascular events was significantly associated to stroke risk (Table B in S1 File). In patients on VKA no difference in TTR was recorded in relation to thrombotic events. Incidence rates of events during follow-up were substantially similar when all DOAC-treated or only DOAC- naïve patients were assessed.

Among patients on DOACs, 378/464 (81.5%) were on treatment with the low-dose of the prescribed drug; furthermore, in 95 of them (20.5%) the reduced dose prescription was not consistent with the recommended dosage in relation to the clinical characteristics of the patients. However, no thrombotic events occurred in the latter subgroup of under-treated patients.

**Fig 1:**
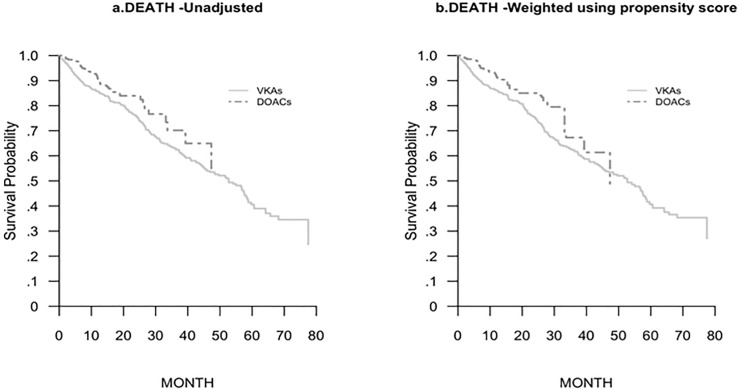
Kaplan Meier curves of mortality unadjusted (a) and weighted using propensity score (b).

A survival analysis, using the Cox proportional hazard model for major bleeding, thrombotic events and death in patients on VKAs and DOACs naïve, is reported in Table 3. Mortality rate was lower in patients on DOACs with respect to those on VKAs, with a HR of 0.64 (95% CI 0.46–0.91) at the multivariate Cox proportional hazard model (Table 3, Fig 1).

**Table 3:** Patients with atrial fibrillation, treatment-naive, 85 years or older. Survival analysis—Cox proportional hazard model, hazard ratio and 95% confidence interval for major bleeding, stroke and death.

			Univariate		Propensity Score Weighted	
		N	HR	95% CI	HR	95% CI
**Major bleeding**	*VKAs*	31/660	1.00	Ref.	1.00	Ref.
	*DOACs*	11/322	0.99	0.50;1.97	0.88	0.42;1.80
**Stroke/TIA**	*VKAs*	8/660	1.00	Ref.	1.00	Ref.
	*DOACs*	8/322	3.24	1.25;8.40	4.04	1.60; 10.20
**Death**	*VKAs*	224/660	1.00	Ref.	1.00	Ref.
	*DOACs*	42/322	0.67	0.48;0.94	0.64	0.46;0.91

VKA = vitamin k antagonist; DOAC = direct oral anticoagulant; TIA = transient ischemic attack

The competing risk analysis confirmed this finding, and showed a significant association of death with coronary artery disease, renal failure, COPD and frailty (Table C in S1 File) Patients on VKAs who died had a lower TTR (median 65%, IQR 52–73) with respect to survived patients (median 72%, IQR 63–80), p = 0.001

## Discussion

The main results of this observational, prospective cohort study of very old AF patients on anticoagulant treatment with either VKAs or DOACs, may be synthesized as follows. There were a few but clinically relevant differences between the baseline characteristics of patients treated with VKAs or DOACs; the incidence of bleeding complications was similar between the two groups, whereas more thrombotic events occurred in patients treated with DOACs; finally, mortality was significantly higher among patients receiving VKAs.

Many baseline characteristics of patients in the two groups were similar, including the median values of bleeding and thrombotic risk scores (HAS-BLED and CHA_2_DS_2_VASc), others showed differences. In some cases the difference was not surprising; is this the case for the lower number of patients with severe renal impairment treated with DOACs, in line with the recommendation against their use in this clinical condition by current guidelines [20]. We found a markedly higher prevalence of coronary artery disease (CAD) among VKAs patients; an observation which is in line with the results of previous observational studies on the general AF population in whom a higher proportion of patients with CAD in VKA treated patients was also found [21]. This may reflect initial concerns derived from the RE-LY study in which a small numerical increase in myocardial infarction in dabigatran patients was detected, and the 2012 European Society of Cardiology (ESC) recommendations to prefer VKAs over DOACs in patients on concomitant antiplatelet therapy [22, 23]. Nowadays, the evidence changed, and the recent ESC guidelines indicate that the bleeding risk seems to be lower with a DOAC plus antiplatelet combination than with a VKA plus antiplatelet combination [24]. In this study, patients on DOACs showed a higher prevalence of previous stroke with respect to patients on VKAs. This finding is probably related to the expected higher efficacy of these drugs in stroke prevention that oriented the treatment choice.

In our cohort the rate of bleeding events was similar between the two treatment groups. In relation to risk factors for bleeding, patients on DOACs showed a higher prevalence of history of bleeding, that is a known risk factor for recurrent bleeding [8]. However, other major bleeding risk factors, such as renal failure or CAD, were more common in VKA patients, even if these data were not statistically significant at competing risk analysis. Surprisingly, we could not detect a benefit for DOACs in terms of occurrence of intracranial hemorrhage. This finding is in contrast with data widely reported in previous studies [2, 4] and may be, at least in part, due to the older age of our cohort. Conversely, the higher rate of gastrointestinal bleeding in DOACs patients is in keeping with previous published data [2, 4, 21].

In the present study patients treated with DOACs had a higher rate of thromboembolic events during anticoagulation in comparison to VKAs patients. As mentioned above, patients on DOACs had at inclusion a higher prevalence of previous stroke with respect to patients on VKAs. This prescription decision may have led to a selected cohort of patients at increased risk for stroke recurrence and may explain, at least in part, the higher rate of thromboembolic complications; it is well known, in fact, that the history of previous stroke is a strong risk factor for recurrence [25]. At partial confirm of this interpretation, it can be added that at the competing risk analysis conducted in our cohort, there was a trend, without statistically significance, for an association between history of previous stroke and occurrence of stroke while on treatment. Another possible explanation for the increased rates of thrombotic events in the DOACs cohort is in the high rate (80%) of DOACs patients treated with the lower dosage of each drug. However, for most patients, this dose reduction was in line with the recommendations of the regulatory agencies. Moreover, no cerebral ischemic events while on treatment were recorded in patients receiving the reduced dose of the DOACs without having the correct indication. Rather, we cannot exclude that a reduced adherence to treatment could also be responsible for this finding, since it is well known that a good adherence to treatment with DOACs is not easy to warrant and is also hard to be evaluated in real life [26]. Conversely, patients on VKAs carefully followed by experienced anticoagulation clinics showed a good quality of treatment, as indicated by a median TTR of 70%, thus suggesting a good clinical control by the participating centers.

An important finding of our study is the higher mortality rate recorded in patients treated with VKAs with respect to those receiving DOACs. This result is in keeping with previous results reported either in the randomized trials [2] and in retrospective real-life data [21], and also related to elderly patients [4]. Certainly, **i**n our study, patients with CAD or renal failure were much more prevalent among those treated with VKAs, both conditions associated with an increased risk for mortality. Furthermore, we could detect a strong and significant association between the presence of these conditions with mortality. We do believe, however, that the higher prevalence of these serious conditions may only partially explain the higher mortality rate recorded among VKAs patients; the lower mortality can better be considered a “class” effect of DOACs.

### Limitations of the study

This study has a number of limitations. Firstly, the observational design requires extreme caution in interpreting direct comparisons between drugs due the intrinsic limitations and high risks of bias of these studies. Secondly, frailty was defined by using clinical items for dementia, bed rest and frequent falls that could be easily reported by the investigators, without the use of a validated, structured frailty stratification score. This explains the relatively low rate of the indicated items recorded in our cohort, where only very severe frailties were recorded. Third, the causes of death reported in the electronic files aimed to identify deaths related to bleeding events or cerebral ischemic events. All other causes of death have been defined as not related to anticoagulant treatment, including cancer, infectious diseases, vascular events (not cerebral), heart failure, renal failure, respiratory insufficiency, or sudden death. Finally, there was no central adjudication of outcome events in this study.

Strengths of our study include the prospective design, the large cohort of very elderly patients, the duration and the high quality of follow-up, performed by experienced centers where patients are routinely closely monitored. This strength is supported by the very low rate of patients lost to follow-up and also by the high persistence on both treatments.

## Conclusion

In conclusions, the results of this prospective observational cohort study, conducted on very elderly AF patients, confirm the overall safety and effectiveness of anticoagulant treatment in this population. Patients treated with DOACs had a lower mortality rate than those receiving VKA, independently of the baseline clinical characteristics, while had a higher risk for cerebral thrombotic events. The bleeding risk was similar in the two groups of treatment.

## Supporting information

S1 File Table A: Survival analysis—Fine and Gray competing risk model, hazard ratio and 95% confidence interval- Major Bleeding. Table B: Survival analysis—Fine and Gray competing risk model, hazard ratio and 95% confidence interval- stroke. Table C: Survival analysis—Cox proportional hazard model, hazard ratio and 95% confidence interval- Death Propensity score single factors.
(DOCX)
