# Supplementary material for: Oral anticoagulation in very elderly patients with atrial fibrillation: Results from the prospective multicenter START2-REGISTER study
Source: PLoS One. 2019 May 23;14(5):e0216831. doi: 10.1371/journal.pone.0216831 (PMC6532867; doi:10.1371/journal.pone.0216831)
Supplement: S1 File — Table A: Survival analysis—Fine and Gray competing risk model, hazard ratio and 95% confidence interval- Major Bleeding. Table B: Survival analysis—Fine and Gray competing risk model, hazard ratio and 95% confidence interval- stroke. Table C: Survival analysis—Cox proportional hazard model, hazard ratio and 95% confidence interval- Death Propensity score single factors. (DOCX) [file pone.0216831.s001.docx]

**Supporting tables**

**S1 Table A**

Survival analysis - Fine and Gray competing risk model, hazard ratio and 95% confidence interval- Major Bleeding

|  |  | **OVERALL** | | | |
| --- | --- | --- | --- | --- | --- |
|  |  | **Univariate** | | **Multivariate** | |
|  |  | **HR** | **95% CI** | **HR** | **95% CI** |
|  |  |  |  |  |  |
| **Treatment** | *VKAs* | 1.00 | Ref. | 1.00 | Ref. |
|  | *DOACs* | 0.99 | 0.50;1.97 | 0.90 | 0.42;1.91 |
| **Age at enrollment** | *(years)* | 1.05 | 0.94;1.16 | 1.06 | 0.95;1.18 |
| **Sex** | Male | 1.00 | Ref. | 1.00 | Ref. |
|  | Female | 0.60 | 0.33;1.10 | 0.67 | 0.35;1.26 |
| **Diabetes mellitus** | No | 1.00 | Ref. | 1.00 | Ref. |
|  | Yes | 1.36 | 0.65;2.83 | 1.27 | 0.61;2.66 |
| **Hypertension** | No | 1.00 | Ref. | 1.00 | Ref. |
|  | Yes | 0.91 | 0.41;2.05 | 0.87 | 0.38;1.95 |
| **Frail subjects** | No | 1.00 | Ref. | 1.00 | Ref. |
|  | Yes | 0.78 | 0.24;2.55 | 0.72 | 0.21;2.51 |
| **COPD** | No | 1.00 | Ref. | 1.00 | Ref. |
|  | Yes | 1.21 | 0.54;2.73 | 1.00 | 0.44;2.25 |
| **Previous bleeding** | No | 1.00 | Ref. | 1.00 | Ref. |
|  | Yes | 4.11 | 1.62;10.39 | 3.40 | 1.17;9.89 |
| **Previous stroke** | No | 1.00 | Ref. | 1.00 | Ref. |
|  | Yes | 1.74 | 0.90;3.39 | 1.66 | 0.78;3.56 |
| **Active cancer** | No | 1.00 | Ref. | 1.00 | Ref. |
|  | Yes | 5.05 | 1.76;14.50 | 5.03 | 1.73;14.66 |
| **Renal Failure** | No | 1.00 | Ref. | 1.00 | Ref. |
|  | Yes | 0.89 | 0.40;1.99 | 1.03 | 0.43;2.45 |
| **Coronary artery disease** | No | 1.00 | Ref. | 1.00 | Ref. |
|  | Yes | 1.34 | 0.66;2.72 | 1.12 | 0.50;2.52 |

**S1 Table B**

Survival analysis - Fine and Gray competing risk model, hazard ratio and 95% confidence interval- stroke

|  |  | **OVERALL** | | | |
| --- | --- | --- | --- | --- | --- |
|  |  | **Univariate** | | **Multivariate** | |
|  |  | **HR** | **95% CI** | **HR** | **95% CI** |
|  |  |  |  |  |  |
| **Treatment** | *VKAs* | 1.00 | Ref. | 1.00 | Ref. |
|  | *DOACs* | 3.24 | 1.25;8.40 | 4.05 | 1.37;12.01 |
| **Age at enrollment** | *(years)* | 1.07 | 0.92;1.24 | 1.03 | 0.88;1.20 |
| **Sex** | Male | 1.00 | Ref. | 1.00 | Ref. |
|  | Female | 0.48 | 0.18;1.30 | 0.42 | 0.16;1.08 |
| **Diabetes mellitus** | No | 1.00 | Ref. | 1.00 | Ref. |
|  | Yes | 1.68 | 0.55;5.17 | 1.26 | 0.39;4.08 |
| **Hypertension** | No | 1.00 | Ref. | 1.00 | Ref. |
|  | Yes | 0.62 | 0.21;1.78 | 0.75 | 0.25;2.23 |
| **Frail subjects** | No | 1.00 | Ref. | 1.00 | Ref. |
|  | Yes | 2.41 | 0.69;8.38 | 2.35 | 0.66;8.43 |
| **COPD** | No | 1.00 | Ref. | 1.00 | Ref. |
|  | Yes | 0.41 | 0.05;3.13 | 0.26 | 0.02;3.06 |
| **Previous bleeding** | No | 1.00 | Ref. | 1.00 | Ref. |
|  | Yes | NE | - | NE | - |
| **Previous stroke** | No | 1.00 | Ref. | 1.00 | Ref. |
|  | Yes | 2.02 | 0.71;5.70 | 2.02 | 0.67;6.11 |
| **Active cancer** | No | 1.00 | Ref. | 1.00 | Ref. |
|  | Yes | 3.16 | 0.45;22.42 | 6.84 | 0.66;71.2 |
| **Renal Failure** | No | 1.00 | Ref. | 1.00 | Ref. |
|  | Yes | 1.43 | 0.49;4.16 | 2.44 | 0.62;9.53 |
| **Coronary artery disease** | No | 1.00 | Ref. | 1.00 | Ref. |
|  | Yes | 2.56 | 0.94;6.99 | 2.82 | 0.98;8.09 |

**S1 Table C**

Survival analysis - Cox proportional hazard model, hazard ratio and 95% confidence interval- Death Propensity score single factors

|  |  | **OVERALL** | | | |
| --- | --- | --- | --- | --- | --- |
|  |  | **Univariate** | | **Multivariate** | |
|  |  | **HR** | **95% CI** | **HR** | **95% CI** |
|  |  |  |  |  |  |
| **Treatment** | *VKAs* | 1.00 | Ref. | 1.00 | Ref. |
|  | *DOACs* | 0.67 | 0.48;0.94 | 0.63 | 0.44;.89 |
| **Age at enrollment** | *(years)* | 1.12 | 1.07;1.17 | 1.13 | 1.08;1.18 |
| **Sex** | Male | 1.00 | Ref. | 1.00 | Ref. |
|  | Female | 0.80 | 0.63;1.01 | 0.76 | 0.59;0.98 |
| **Diabetes mellitus** | No | 1.00 | Ref. | 1.00 | Ref. |
|  | Yes | 0.99 | 0.72;1.37 | 0.90 | 0.64;1.24 |
| **Hypertension** | No | 1.00 | Ref. | 1.00 | Ref. |
|  | Yes | 0.82 | 0.59;1.12 | 0.75 | 0.55;1.02 |
| **Frail subjects** | No | 1.00 | Ref. | 1.00 | Ref. |
|  | Yes | 1.79 | 1.25;2.57 | 1.69 | 1.16;2.45 |
| **COPD** | No | 1.00 | Ref. | 1.00 | Ref. |
|  | Yes | 1.66 | 1.23;2.24 | 1.38 | 1.01;1.87 |
| **Previous bleeding** | No | 1.00 | Ref. | 1.00 | Ref. |
|  | Yes | 1.24 | 0.63;2.45 | 1.14 | 0.56;2.32 |
| **Previous stroke** | No | 1.00 | Ref. | 1.00 | Ref. |
|  | Yes | 1.22 | 0.91;1.64 | 1.31 | 0.95;1.81 |
| **Active cancer** | No | 1.00 | Ref. | 1.00 | Ref. |
|  | Yes | 1.81 | 0.91;3.60 | 1.58 | 0.80;3.13 |
| **Renal Failure** | No | 1.00 | Ref. | 1.00 | Ref. |
|  | Yes | 1.92 | 1.48; 2.48 | 1.86 | 1.41;2.47 |
| **Coronary artery disease** | No | 1.00 | Ref. | 1.00 | Ref. |
|  | Yes | 1.77 | 1.35;2.31 | 1.68 | 1.28;2.21 |
